# The Importance of a Multi-Disciplinary Approach to the Endometriotic Patients: The Relationship between Endometriosis and Psychic Vulnerability

**DOI:** 10.3390/jcm10081616

**Published:** 2021-04-10

**Authors:** Manuel Glauco Carbone, Giovanni Campo, Enrico Papaleo, Donatella Marazziti, Icro Maremmani

**Affiliations:** 1 PISA-School of Experimental and Clinical Psychiatry, 56100 Pisa, Italy; manuelglaucocarbone@gmail.com; 2 Department of Medicine and Surgery, Division of Psychiatry, University of Insubria, 21100 Varese, Italy; 3 Obstetrics and Gynecology Department, IRCCS San Raffaele Scientific Institute, Vita-Salute San Raffaele University, 20132 Milan, Italy; campo.giovanni@hotmail.it (G.C.); papaleo.enrico@hsr.it (E.P.); 4 Reproductive Sciences Laboratory, Division of Genetics and Cell Biology, IRCCS San Raffaele Scientific Institute, Vita-Salute San Raffaele University, 20132 Milan, Italy; 5 1st Psychiatric Unit, Department of Clinical and Experimental Medicine, Santa Chiara University Hospital, University of Pisa, 56100 Pisa, Italy; dmarazzi@psico.med.unipi.it; 6 Saint Camillus International University of Health and Medical Sciences-UniCamillus, 00131 Rome, Italy; 7 Association for the Application of Neuroscientific Knowledge to Social Aims (AU-CNS), 55045 Pietrasanta, Lucca, Italy; 8 Vincent P. Dole Dual Disorder Unit, 2nd Psychiatric Unit, Santa Chiara University Hospital, University of Pisa, 56100 Pisa, Italy; 9 G. De Lisio Institute of Behavioral Sciences, 56100 Pisa, Italy

**Keywords:** endometriosis, bipolar disorder, affective disorder, personality traits, emotional dysregulation, vulnerability, dual disorders

## Abstract

Endometriosis is a chronic inflammatory condition, which is distinguished by the presence of the endometrial-like glands and stroma outside the uterine cavity. Pain and infertility are the most commonly expressed symptoms, occurring in 60% and 40% of cases, respectively. Women with endometriosis, especially those with pelvic pain, also have a greater vulnerability to several psychiatric disorders. There is, in particular, a tendency to contract affective or anxiety disorders as well as panic-agoraphobic and substance use disorders. Endometriosis with pelvic pain, infertility and psychic vulnerability usually leads to disability and a markedly lower quality of life for women of reproductive age. Thus, the burden of endometriosis is not limited to the symptoms and dysfunctions of the disease; it extends to the social, working and emotional spheres, leading to a severe impairment of global functioning. An analysis of scientific literature revealed a close relationship between specific temperamental traits, the expression of several psychiatric symptoms, chronicity of pain, risk of substance use and lower probability of a positive outcome. Endometriosis symptoms and the impact of related psychological consequences, increased vulnerability and the possible onset of psychiatric symptoms may influence coping strategies and weaken resilience, so triggering a vicious cycle leading to a marked deterioration in the quality of life. A multidisciplinary approach consisting of a medical team composed of gynecologists, psychologists, psychiatrists, experts in Dual Disorder, algologists and sexologists, would guarantee the setting of a target and taking the best decision on a personalized treatment plan. That approach would allow the prompt detection of any psychopathological symptoms and improve the endometriosis-related physical symptoms, bringing a healthier quality of life and a greater likelihood of a positive outcome.

## 1. Introduction

### 1.1. Endometriosis

Endometriosis is a chronic and progressive disorder with multifactorial etiopathogenesis, characterized by the anomalous presence of endometrial-like glands and stroma outside the uterine cavity, resulting in an estrogen-dependent chronic inflammatory reaction [1,2,3]. The ectopic endometrium is usually found on the pelvic peritoneum and in the pelvic organs, and it is affected by lesions proximal to the uterus such as the ovaries, salpinges, the ligaments of the cervix and uterus, and the surrounding pelvic peritoneum defining what is often called pelvic endometriosis. Endometriosis also arises in organs and tissues outside or far from the pelvis, including the vagina, vulva, cervix and perineum, the urinary system, the gastrointestinal tract, the thoracic cavity, including the lungs and pleura, the extremities, skin, and central nervous system (CNS), all of which are involved in what is usually called extra-pelvic endometriosis [2,4]. In any case, the term ‘extragenital pelvic endometriosis’ is the one that most accurately describes endometriotic lesions that damage pelvic organs such as the rectum, sigmoid and urinary bladder [5]. When found in association, pelvic and extra-pelvic endometriosis are the defining features of external endometriosis, which accounts for as many as approximately 90–95% of cases of this type [6,7]. In the remaining cases, the ectopic endometrium is alternatively located in the context of the myometrium, thus determining internal endometriosis or adenomyosis [2,4,8]. It has been suggested that adenomyosis might derive from the infiltration of basal endometrium into myometrial dehiscencies [9]. From one point of view, such damage could be due to a process of chronic proliferation and inflammation induced at the level of the endometrial-subendometrial unit or archimetra by chronic uterine auto-traumatization and leading to tissue injury and repairing (TIAR) [10,11,12].

The ectopic endometrium is affected, like the normal uterine mucosa, by stimuli from ovarian hormones, particularly estrogens, thus taking on proliferative and functional (including flaking and bleeding in the menstrual period) comparable to those that occur in the typical endometrium. Endometriosis is therefore considered a disorder mainly of women of reproductive age, affecting about 6–10% of the female population and reaching a peak of incidence between 30 and 40 years in nulliparous women [8,13]. Most women report symptoms since adolescence, and there are rare cases of endometriosis in premenarchal age patients [14,15,16]. Although the incidence and recurrence of endometriosis in postmenopausal women is not fully understood, it would seem to tend to regress in post-menopause or after hysterectomy [13]. The data available on prevalence are strongly conditioned both because of the presence of asymptomatic forms and the existence of microscopic or sub-peritoneal endometrioses that are rarely diagnosed.

Even though diagnosis delay has shortened over the last years, the diagnosis of endometriosis still typically occurs 7–10 years after the onset of the first symptoms [17,18,19,20,21,22]. This is due to the complexity of the etiopathogenesis and the variety of the symptoms that are found, as well as the lack of timely investigation able to rely on non-invasive diagnostic tools [8,23]. Endometriosis is usually diagnosed during laparoscopy, or during a laparotomy performed for another indication followed by the histological examination of the lesions. The localization and extent of the illness can, however, sometimes be determined through an accurate clinical evaluation [24,25,26]. Vaginal and rectovaginal digital examination can facilitate the investigation of pelvic trigger pain points and menstruation usually improves the clinical significance that can be detected [25,27,28,29]. Generally speaking, a common clinical characteristic that arises from pelvic adherences, is what is often called “frozen pelvis” [30].

Many tests utilizing pelvic imaging, eutopic endometrium characteristics, blood and urinary markers or peritoneal fluid components have been proposed as diagnostic elements for endometriosis.

Some diagnostic imaging techniques are very helpful tools to be used for diagnostic purposes in patients with suspected symptoms of endometriosis. The first-line investigation, in this regard, is transvaginal sonography (TVS). This technique is cheap but user dependent. Magnetic resonance imaging (MRI) is a non-invasive procedure that is considerably more expensive and theoretically more precise. Recently, some studies have shown that with the progressive definition improvement (advanced ultrasound), the diagnostic performance of TVS and MRI is similar for detecting deep infiltrating endometriosis (DIE) involving rectosigmoid, uterosacral ligaments and rectovaginal septum [31,32,33]. Many biomarkers have been studied but none consistently met the criteria for a replacement diagnostic test. Some plasmatic biomarkers could be useful for discriminating ovarian endometrioma from other benign ovarian lesions, but there is not enough evidence to draw definitive conclusions [34]. Cancer Antigen 125 or Carbohydrate Antigen 125 (CA-125) is the most used plasmatic biomarker in clinical setting in order to complete and support the diagnostic investigation, but nevertheless it has a low specificity and sensitivity. Looking ahead, a promising diagnostic option could be the use of specific microRNAs (miRNAs) [35,36].

The pathophysiological mechanisms underlying the formation and progression of endometriosis are still unknown. Many theories have been postulated without necessarily excluding each other. The etiology of endometriosis is likely to be multifactorial, while involving an intricate interplay between several factors [37].

The most widely accepted hypotheses are retrograde menstruation (or tubal reflux of menstrual blood), TIAR, vascular diffusion, metaplasia of coelomic epithelial origin, apoptotic/proliferative pathways, embryogenesis disturbances, genetic predisposition, alterations of the immune system, hormonal dysfunctions and surgical dissemination [9,38,39,40]. Equally, there are various and shaded endometriosis-related clinical situations or symptoms, and they generally depend on the localization of the lesions. Though diarrhea or constipation, often with dyschezia and/or hematochezia, are frequent signs of intestinal involvement; dysuria and hematuria indicate a possible urinary implication; vomiting, nausea, headaches are usually pain-related [41,42,43,44,45,46]. 

### 1.2. Pain

Women with endometriosis, however, usually report pelvic or abdominal pain, which occurs in about 60% of these cases, and infertility. About a third of infertile women are affected by endometriosis, and about 40% of women with endometriosis have fertility problems [13,47]. It could be said that infertility is a common but not a specific feature of these patients; therefore physicians, during a medical consultation for sterility, should have the suspect of the presence of an endometriotic lesions, due to the evocativeness physical examinations, TVS signs and the known association between these disorders. 

Pain is the common thread of all the clinical endometriotic situations, and it can be fulfilled in different ways, depending on lesion localizations and temporary. So, they can be represented by dysuria, dyschezia, dysmenorrhea, dyspareunia, cyclic and non-cyclic pain. If dysuria and dyschezia represent respectively the pain manifestations of endometriotic lesion in urinary system and intestinal tract, dysmenorrhea and dyspareunia are common pain manifestations of a DIE [48,49]. As far as temporary goes the pain firstly cyclic and menstruation related, became non- or acyclic due to inflammation, strong visceral adherences generation and nervous sensibilization until became after the duration of six months chronic pelvic pain (CPP) [50,51]. 

Dysmenorrhea is described as pelvic pain associated with menstrual flow, while deep dyspareunia is pelvic pain during deep sexual penetration [45]. CPP is one of the main features of the endometriosis symptomatology [52]. CPP is defined as pain that occurs in the pelvic area (below the umbilicus) and lasts for at least six months, while being severe enough to cause functional disability or require medical or, in many cases, surgical treatment [53,54]. It may or may not be associated with periods. While CPP can be a symptom caused by one or more different conditions, it often proves to be a chronic condition related to how the central nervous system processes threat perception (“central sensitization”) [55]. Central sensitization is a condition of the CNS that is closely associated with the development and maintenance of chronic pain. When central sensitization occurs, the nervous system goes through a specific process called ‘wind-up’, so that it becomes set in a persistent state of high responsiveness. This regulated, ongoing state of reactivity drops the threshold for all possible causes of pain, and subsequently takes on the role of maintaining pain even after the initial injury might have healed [56,57,58,59]. 

The impact of pain is dynamic; it is experienced in a subjective and multifaceted way, the comprehension of which necessitates a good description of its features in each single patient [60]. Indeed, it may be reported as occurring every day, once a month, or rarely, and it changes in each patient over time and across life stages [61]. About 25% of affected women are asymptomatic, regardless of the ectopic endometrial tissue dimensions. What Hurd refers to as “perceived” pain seems to be independent of the stage of the disease: women with only mild endometriosis who suffer from disabling painful symptoms can be observed, and vice versa [62].

This finding may suggest that other factors, such as personality traits, emotional and affective factors, coping and behavioral strategies, altered stress reaction, attention, interpretations and beliefs about the pain (i.e., about its duration, controllability and cause) may affect the perception of pain, so influencing its intensity and tolerability [63,64,65,66,67]. Psychological processes of emotions, thought, and behavior involve several neuronal networks rather than distinct centers. Some of these processes are elaborated and others fundamental in evolutionary terms, and their interaction with pain processing is complex. Moreover, these psychophysiological elements seem to play an important role in the development of central sensitization. Several studies have shown the relationship between stress and the lowering of pain thresholds [68,69,70,71]. Likewise, a previous history of mood and anxiety spectrum disorders, as well as physical and psychological traumas, are significantly predictive of developing central sensitization and have the capacity to make pain chronic [72,73,74,75,76]. It may be suggested that these psychophysiological factors make subjects more prone to become centrally sensitized once the onset of pain occurs. Endometriosis has a strong impact on self-esteem, affective and emotional stability, as well as on the social and working functioning of affected women, causing distress and a considerable lowering of the quality of life [77,78,79].

Probably, it is the experience of pain, rather than endometriosis itself, that is crucial in causing emotional distress and worsening psychiatric symptoms [80]. It is, in any case, a hard task to distinct these aspects (endometriosis itself and pain), since they influence each other shared mechanisms, such as neural and immune-oxidative ones [81,82].

### 1.3. Neuropsychiatric Symptoms

Despite the important potential psychosocial implications of endometriosis, there is a lack of research concerning the relationship between endometriosis and neuropsychiatric symptoms, as well as the possible mechanisms underlying this relationship.

The scientific literature shows that endometriosis is widely associated with a high rate of psychiatric symptoms. Statistically significant associations were found between endometriosis, personological traits, affective disorders, anxiety disorders, substance use disorder and other psychiatric diseases [83,84,85,86,87,88,89,90,91,92,93,94,95,96] (See Table 1).

### 1.4. Aim of This Narrative Review

We analyzed the scientific literature investigating the relationship between neuropsychiatric elements and endometriosis in order to detect useful clinical information to the medical staff and set up a personalized multidisciplinary treatment for patients with endometriosis. The authors of the present paper consider fundamental a multidisciplinary approach to the endometriotic patient, through the help of a medical team comprising gynecologists, psychologists, psychiatrists, specialists in Dual Disorder conditions, algologists and sexologists. By working together, this team will be able to promptly identify the possible presence of typical personological features or neuropsychiatric symptoms in order to guarantee a specific integrated personalized treatment.

## 2. Neuropsychiatry and Endometriosis: The State of the Art 

### 2.1. Depressive Disorders and Endometriosis

The term “depression” defines a broad group of psychopathological diseases characterized by the presence of depressed or irritable mood, cognitive and somatic symptoms that induce the maladjustment of work, family, or educational life, eating habits, sleep and physical health, with a progressive impairment of quality of life [97,98]. Based on the cognitive model of depression, it is fair to propose that depression results from the interaction between the predisposed factors of each patient, their cognitive structures, the various stress factors, the disease, and its resulting symptoms [99,100,101].

According to DSM-5 [97], depressive disorders include: Major Depressive Disorder (MDD), Persistent Depressive Disorder or Dysthymia (PDD), Premenstrual Dysphoric Disorder (PMDD), Disruptive Mood Dysregulation Disorder (DMDD), Substance or Medication Induced Depressive Disorder, Other Specified Depressive Disorders and Depressive Disorders Not Otherwise Specified (DD-NOS). Unfortunately, most of the selected studies did not use clinical diagnostic criteria to be assessed in formulating a psychiatric diagnosis. To meet this need, we also report below some studies, which, although not using appropriate and specific diagnostic means, identified symptoms belonging to the depressive spectrum in endometriotic patients.

Starting from the clinical observation that several women with neuropsychiatric symptoms seen in psychiatric practice had been diagnosed by their gynecologists as suffering from endometriosis, Lewis et al. (1987) wondered if there might be an association between psychiatric illness and endometriosis. They found a high prevalence of mood disorders in endometriotic patients speculating about a possible hormonal or neurotransmitter link as a possible cause. The findings of this study, were, however, strongly limited due to the lack of a longitudinal evaluation of the patients, the absence of a control group and psychiatrists who evaluated the patients were not blind to the results of laparoscopy, so this could have biased their findings [102]. 

Waller et al. (1995), after recruiting a total sample of 118 subjects underwent to laparoscopy for pelvic pain symptoms, infertility, or sterilization, found that the severity of depression (assessed by Beck Depression Inventory—BDI—[103]) is related to chronic pain, regardless of the presence of endometriosis. The lack of a longitudinal evaluation and a psychiatric examination as well as the use of self-reported rating scales may have limited the value of the findings of the study [104]. Similarly, the high prevalence of depressive symptoms in symptomatic endometriotic women, was confirmed by Lorencatto et al. (2006) [105] that, using BDI, detected depressive symptoms in 86% and 38% in a sample of endometriotic women with and without CPP, respectively. Somatic concern, work inhibition, dissatisfaction, sense of failure and melancholy were the depressive complaints with the greatest discriminatory power between the two groups. Furthermore, the authors noticed that the hormonal therapy did not influence the affective dimension, although some reports describe a significant relationship between the use of hormonal medication and the occurrence of depressive symptoms [106,107]. These findings need to be seen in light of some limits: the authors did not evaluate the time lapse between the initial diagnosis of endometriosis and the consequent admission to the study because longer periods of pain could lead to higher degrees of depression. Furthermore, pain was evaluated as a dual variable; henceforth, several levels of pain scores were not individualized to permit an association between the degree of pain and the depression index [105]. The same research group, in a different study, observed that psychological support (group intervention) positively impacts the psycho-affective well-being of patients with endometriosis, both reducing depressive symptoms and improving pain management. A positive correlation was further identified between pain and depression severity [108]. The use of the BDI as a diagnostic scale for depression and the Visual analogue scale (VAS) to measure pain intensity partially limit the results of this study. Generally, pain measurement using scales has limitations, as this symptom is a multifactorial subjective manifestation influenced by the person’s emotional state. Furthermore, with VAS patients have difficulty finding the point on the line that best applies to them, i. e., weighing up the significance a distance from the verbal anchor has. 

Siedentopf et al. (2008), in a sample of women underwent laparoscopy because of unexplained infertility, found that women with endometriosis reported a fall in quality of life, increased stress perception and depressive symptoms, particularly those at an advanced stage. In the meanwhile, in an attempt to identify an “immunological fingerprint” for endometriosis in serum by analyzing cytokine levels, they noticed how Th1⁄ Th2 ratio was in favor of Th1, accompanied by the increased levels of IFN-α, which unveiled a shift towards the pro-inflammation but only in early stages of endometriosis [109]. 

Other authors identified a significant association between age and depressive symptoms suggesting that staging/extent of endometriosis could be related with the increase of the severity of the emotional suffering caused by the disease, which manifests as depressive symptoms [110]. In this case, the findings could be flawed because the patient sample might not be representative of the whole endometriotic population, since the study was conducted in referral centers that typically treat more complex cases with CPP. Differently, in a sample of fifty-seven patients, underwent laparoscopy because CPP, was found a high prevalence of depressive and anxiety symptoms, independently of an endometriosis diagnosis. The absence of a disease-free or pain-free control group, the lack of a psychiatric clinical examination, as well as the use of self-reported instruments to detect psychiatric symptoms, stand as the main limitations of the study [80].

Kumar et al. (2010) showed that patients with CPP had higher scores in depression, alienation and impairment in quality of life compared to both healthy and endometriotic women [111]. Some limitations should be considered: no longitudinal evaluation, no complete anamnestic collection performed by psychiatrists and, above all, the authors did not specify if there were symptoms in women with endometriosis, so we are unable to weigh their possible impact [112]. Subsequently, in a different study, the same authors (2011) did not find any differences in the prevalence of the depressive disorder between endometriotic and women complaining CPP. Instead, they described a significantly greater proportion of bipolar disorder in the endometriotic group. The major limitations of the study are the small number of patients recruited the heterogeneity between the groups compared, and the lack of a longitudinal evaluation. The Symptomatological set of patients with endometriosis was not reported either. Furthermore, in the opinion of the authors, another limit to be considered is that, as the study was performed in a specialized center for women with endometriosis, it is quite feasible that the women who find themselves in this kind of setting mostly have a chronic or more debilitating form of the disease, thus reducing the potential for generalizing of the results [113].

A clinical evaluation of the mental status of 50 patients with endometriosis detected depressive symptoms in the majority of the sample (78%). These women complained of sleep disorders, asthenic manifestations (reduced working capacity, rapid fatigue, disorders in concentration of attention and memory) and, further, they clearly described a worsening of depressive manifestations in relation to the severity degree of painful symptoms. As a rule, anxiety and depression were co-morbid in the patients examined. What is more, the authors found a positive correlation between severity of depressive symptoms and IL-1β, IL-2, IFN-γ levels, resulting in the imbalanced production of pro- and anti-inflammatory (IL-4) cytokines [114]. 

A retrospective review of medical records showed that depression and anxiety were also common both in adolescents and young endometriotic women and that the prevalence of affective disorders was even higher in patients with pain syndromes in comorbidity. This study has limits due to its design and to the reliance on patients’ medical records and patients’ self-reported diagnoses for the identification of comorbid pain and affective conditions, without the use of standardized diagnoses. The retrospective nature also curtailed the ability to assess patients’ symptoms. Obviously, this sample may represent a more symptomatic group of endometriosis patients, since they were referred to a tertiary care clinic, which means that the true prevalence of some of these comorbid conditions may be lower in a community-based sample [78].

A preliminary study, performed by Cavaggioni et al. (2014), revealed a statistically significant differences between cases and controls in the prevalence of psychiatric disorders (54% and 18.6%, respectively), particularly, those belonging to the depressive and anxious spectrum. Patients with endometriosis-associated pain presented a greater prevalence of psychiatric disorders compared with pain-free patients, but that difference did not reach the level of significance. Although these findings arouse great interest, the small sample size posed a major limitation, as they did not allow any definitive conclusions to be drawn [83]. Likewise, comparing histologically confirmed ovarian endometriotic patients with patients who had had benign adnexal diseases, it was found that somatization, depression, sensitivity, anxiety levels and phobic anxiety were higher in the endometriosis group than in the control group. The nature of the study design and the diagnostic assessment using self-report scale limited the findings of this study [89]. A defensive response style, biases regarding positive personal traits filtered and subjective rater perceptions, social desirability, and “halo effect”, are all plausible biases of every self-report measure. Finally, they did not consider the duration of the symptoms presented.

On the other hand, endometriotic women, attending an outpatient department for the treatment of fertility-related problems, expressed significantly more catastrophism about their pain, more depression and anxiety symptoms, more frequent pain during intercourse, higher levels of chronic pain, more impairment of the sexual functioning and more impairment of life quality than a control group. Recruitment sites (predominantly tertiary care centers) have probably led to an overrepresentation of patients with a more severe degree of endometriosis. Other limitations were the inadequate diagnostic tools used to detect psychiatric symptoms and the omission of any psychiatric examinations [115].

Chen et al. (2016), using the Taiwan National Health Insurance Research Database, performed the first longitudinal study to investigate the temporal association between endometriosis and depression and anxiety disorders. Women with endometriosis had an elevated risk of developing depressive and anxiety disorders compared to those without endometriosis. Both younger women aged < 40 years and older women aged ≧40 years with endometriosis were prone to developing these disorders. In any case, we should consider that the incidence of depression and anxiety disorders may be under-estimated, as only those who had sought medical consultation qualified for enrolment in the study. The authors did not investigate the association between endometriosis medications and the risk of depression or anxiety disorders, and did not evaluate the impact of dysmenorrhea, dyspareunia or pelvic pain on the risk of psychiatric symptoms [85].

An exploratory study, including women undergoing treatment for endometriosis, determined that the use of positive coping strategies displayed better adaptation to stress and fewer depressive symptoms. In contrast, the use of maladaptive coping strategies focused on emotion is correlated with an increase in depression and stress. The small sample size and the design of the study (for not including a control group) can be named as the major limitations. Furthermore, the institutions in which the research was done is a tertiary referral center for treatment of endometriosis, in a such way, that most of the patients included in the study presented severe degree of the disease, which could be an important factor of biased in the obtained result [116].

The research group of the University of Siena, using the Patient Health Questionnaire (PHQ), a self-administered screening tool for mental health disorders, found that 59% of endometriotic women recruited were affected by at least one psychiatric disorder, showing a significant positive correlation with pain symptoms [117]. As pointed out by the authors, the study is in part limited by the lack of any clinical confirmation of psychiatric disorder and the contribution from a non-profit organization of patients with endometriosis for the recruitment of a study population likely to produce a selection bias [95].

In a sample consisting of 202 women with endometriosis, about 43.1% of the sample reported moderate to severe symptoms of depression. Physical functioning, feelings about the medical profession and sexual relationships were significant predictors of symptoms of depression. In this case, the cross-sectional research design did not allow us to examine the temporal relationships between variables. The participants who followed the route of recruitment through public tertiary hospitals and private infertility clinics are subject to the likelihood that their degree of severity of endometriosis could well be greater than that of the participants who did not request treatment [118].

It should now be pointed out that some studies failed to show any specific predispositions in women with endometriosis to develop either psychiatric symptoms or other serious medical conditions.

As part of a longitudinal study on CPP, Walker et al. (1989) did not find any significant differences between endometriotic women and women without endometriosis in the prevalence of bipolar disorder, major depression, alcohol abuse, history of sexual abuse and CPP. The small size of the sample, as well as the heterogeneity of the groups being compared, limited the value of the findings [119].

Similarly, another cross-sectional study, comparing women with CPP and endometriosis and women with CPP and other gynecological problems, did not find any differences on BDI scores and pain ratings. Nevertheless, at the same time, both groups received neuroticism, anxiety and psychiatric morbidity scores higher than those indicated by general population data. This study presents various methodological limitations. First, patients were never evaluated by a team of psychiatrists, and some of the rating scales such as the BDI and STAI, which are used to assess specific personality traits, affective and anxious symptoms, are self-reported, thus limiting the psychopathological evaluation of the patients. Second, the samples are small and were not matched by age. Lastly, the non-longitudinal nature of the study leaves no scope for definitive conclusions [120]. 

No differences in mood symptoms or personality characteristics were also identified by Peveler et al. (1996), even if women with endometriosis presented more severe pain and greater social dysfunctionality. In this case, the interviews were performed after diagnostic laparoscopy, so some of the patients might have discovered the outcome of their investigations and this knowledge may probably have influenced their responses to the questionnaires; mood symptoms were measured using a self-report questionnaire without any psychiatric clinical examination; in addition, there was no pain-free control group [121].

Also comparing pain-free endometriotic women to asymptomatic women with endometriosis, no significant differences were found between groups on depression/anxiety scores. Significant positive correlations were found between coping and depression/anxiety, and between pain severity and subjective psychosocial impairment. In this case too, a few limitations in methods of assessment and in the lack of control groups were present [66]. Same results were obtained matching women suffering from CPP secondary to endometriosis, myofascial abdominal/pelvic pain or pelvic adhesions. It must, however, be noted that the study under review has several methodological limitations. The sample size was small and derives from a tertiary care setting, which means that the women participating in the study are likely to present with more severe conditions. Further, it was not a quantification of the degree of endometriosis; this serious shortcoming excluded any accurate examination of the extent and severity of endometriosis in relation to various psychological parameters associated with presenting complaints about pain. Given the nature of the study design, all the relationships between the independent and outcome variables are correlational, so quantitative assessment must be limited to determining possible causal relationships between a particular psychological variable and the data on pain outcomes [122]. In a sample of women with persistent postsurgical pain after surgery for endometriosis, depression, as measured by the BDI, was not associated with differences in pain severity, even if the 35.8% of the women on antidepressants reported significantly higher pain intensity. If we admit that the women who have been taking antidepressant therapy were probably those who had greater severity of depression, we could hypothesize a possible link between the degree of depression and the severity of perceived pain. The study under review was limited by its retrospective cross-sectional design and small sample size [123]. In the same way, no significant differences regarding symptoms of depression or anxiety were found between a sample of endometriotic women and a healthy-control sample [124]. The divergence of the results could be partially explained by the symptomatological differences among patients; most of them received a diagnosis of endometriosis because of infertility or following a random finding during diagnostic laparoscopy and not primarily of pain. Duration of fertility treatment or even current life events were not recorded in this study and this may have led to a considerable bias in the interpretation of the data. Nevertheless, other limitations should be considered: the retrospective nature of the study, the small sample size, the absence of a psychiatric clinical examination and the heterogeneity between groups (no age or sociodemographic matching of the control group had been performed). 

Interestingly, an observational prospective study found that the amelioration of the chronic pain in a cohort of 117 subjects of the outpatient services for endometriosis and chronic pelvic pain, independently on treatment (surgical or pharmacological) did not affect the depressive symptomatology. However, the management of the pain during menses, between menses and at intercourse positively influence the quality of life and reduced the anxiety levels assessed by STAI-Y1 [125]. The lack of a psychiatric evaluation and the use of self-rating scales to assess depressive symptoms have probably partially biased these findings.

The current Major Depressive Disorder, as well as the Bipolar Disorder, proved to be more prevalent in women with CPP. Endometriosis was associated with CPP in 48% of the women but, with specific reference to endometriosis, no significant difference was recorded in the prevalence of psychiatric disorders [90]. 

Definitively, women with endometriosis, especially those with the incessant presence of pain, often experience changes in the dynamics of their affective, family, and social life. Initially, the individual experiences the loss of a healthy, active body, then proceeds to a state of dependency and limitations. In addition, a loss of social and economic status may occur because of declines in physical performance. Affective relationships may be impaired too, considering that the person with pain usually gives voice to several complaints. Cognitive perceptions may change and feelings of disability and loss of self-esteem are often observed in such patients; a depressive reaction develops that impairs a patient’s compliance with treatment, repeatedly leading to unsatisfactory results in the struggle to control the disease.

Analysis of the literature allows various non-univocal results to emerge, but some specific trends are easily identified. Most studies unhesitatingly name psychopathological symptoms on the basis of self-reported evaluation forms or online questionnaires. They also report diagnoses of psychiatric pathologies independently of any longitudinal psychiatric clinical evaluation, so making it evident that women with endometriosis who come to the attention of the gynecologist, in particular those who complain of CPP, tend to present symptoms of a depressive nature, and have a history that includes depressive disorders at a greater frequency than non-endometriotic women. Together with the presence of depressive elements, there is a clear decline in the quality of life, accompanied by a higher degree of social and work impairment. Marriage status appears to be a protective element in the development of depressive symptoms and/or disorders. The severity of depression and the degree of sexual satisfaction seem to be connected. In the same way, psychopharmacological and psychotherapeutic treatment, by stimulating the affective component, improve the patient’s quality of life, pain management and overall outcome. 

There is also a compelling need to highlight an everyday occurrence in the clinical setting, which is the improper interpretation of depressive symptoms – a problem leading to the writing of incorrect diagnoses. Some neuropsychiatric disorders, especially, bipolar disorder, are commonly misdiagnosed as unipolar depression, probably because people with an affective disorder often initially undergo a clinical evaluation for the treatment of depression [126,127,128,129]. When they are asked to recall any hypomanic symptoms, they do not name any, either because others do not comment on these symptoms, or because there is little impairment or there may even, occasionally, be an improvement associated with them [129]. Misdiagnosing these symptoms as unipolar depression has important clinical implications. The “false depressed” are often treated exclusively with antidepressant medications, which may lead to a worsening of the course of illness and deprive the patient of the potential benefits of appropriate medications [129]. Ghaemi et al. (2000) stated that 23% of those misdiagnosed with depression experienced a new or worsening, rapid-cycling course attributable to anti-depressant use [126].

### 2.2. Bipolar Disorder and Endometriosis

In recent years, interest in the study of the relationship between Bipolar Disorder (BD) and Endometriosis has been growing. Most studies suggest that endometriosis is a potential risk factor for developing BD. In the previous paragraph we reported how Lewis et al. (1987) described a high prevalence of bipolar disorder in women with endometriosis; in fact, 10 out of 16 of the patients who were being treated by laparotomy for a suspected endometriosis were found to have a bipolar disorder. Furthermore, the authors were the first to speculate about a possible hormonal or neurotransmitter link between the two pathologies [102]. Similarly, as previously noted, Kumar et al (2011) found a significantly greater proportion of women in the endometriosis group with bipolar disorder and a poorer quality of life than in the group of women with pelvic pain that was unrelated to endometriosis [113]. 

More recently, Chen et al. (2019) performed a nationwide, population-based cohort study, in which they found that women with endometriosis were associated with an increased risk of developing bipolar disorder when compared with non-endometriotic controls after adjusting for age, comorbidities and a range of different treatment options. In the present study, some results suggested that hormonal or surgical treatment of endometriotic women seemed to have a limited impact on the risk of developing BD, although some treatment options for endometriosis have been associated with emotional instability or a higher risk of BD development. Several limitations of the study should be noted: lifestyle, socioeconomic status and family history were overlooked, medical and psychiatric comorbidities were not taken into account, and the severity of endometriosis in the sample was not assessed. What is more, it was not found to be possible to study the impact on the risk of developing BD for each of the different types of treatment that were assessed [86].

Similarly, in another longitudinal follow-up study, hysterectomy was associated with higher risk of developing BD, especially in women with endometriosis, and those using Premarin (conjugated estrogen) as treatment. Despite all its merits, some weaknesses in the study must be noted. No data on quality of life, education, occupation, marriage status, or even substance use was available for analysis. The diagnosis of bipolar disorder was identified using ICD codes in administrative claims, so raising the risk that the presence of BD may have been underestimated, because only those seeking medical help could be identified in this study. In the database, no information regarding family history in cases of bipolar disorder or other psychiatric illnesses was provided [93].

From a different standpoint, Walker et al. (1989) did not identify statistically significant differences in the onset of BD between women with symptomatic as opposed to asymptomatic mild endometriosis [119]. 

Even in this case, despite the equivocal results, it is possible to identify a specific trend. It would seem that women with endometriosis, especially those with the greatest degree of pain-related experiences, tend to develop greater reactivity to vital events and, therefore, to present a higher risk for mood swings in both polarities, in so far as some authors have considered endometriosis as a risk factor for the development of bipolar disorder. In the meanwhile, we should consider that comorbid bipolar disorder could influence the effects of endometriosis on psychological well-being and on quality of life, besides influencing the capacity to manage pain. These considerations might have important therapeutic implications. What is crucial is to be able to identify any presence of bipolar disorder as early as possible in order to treat and stabilize these subjects, so making them less vulnerable.

Antidepressants have been recommended as adjuncts in the management of CPP, but in people with bipolar disorder they could increase the risk of manic switch, in this way leading to an acceleration of cycle frequency and raising suicidality. It is essential to rule out bipolar disorder before the initiation of antidepressants, thus allowing specialists to weigh up the merits of introducing mood stabilizers in therapy. Moreover, BD usually showed a high frequency of comorbidities, especially with substance use disorder [130]. Its co-occurrence makes it more pernicious by creating difficulties in treating the whole course of illness. It could delay recovery from mood episodes, increase suicidality and functional impairment, while lowering adherence to medications and damaging the quality of life [131,132,133,134,135]. 

Genetic and family studies have likewise suggested a considerable overlap and interaction between the two disorders, which raises the likelihood probability that early onset bipolar disorder and substance abuse disorder share at least some common genetic vulnerabilities [136]. People with BD are more exposed to substance use disorders (SUDs: non-medical use of, or dependence on drugs and/or alcohol), while, viewed from the opposite perspective, a history of SUDs is associated with an earlier onset of BD [131,137]. People diagnosed with BD are more susceptible to a greater use of cocaine, amphetamines, opiates, cannabinoids and hallucinogens, in comparison with other psychiatric disorders such as schizophrenia [138,139,140,141,142,143]. Therefore, endometriotic patients with BD constitute a specific subpopulation of patients that deserves greater attention, especially when it comes to deciding on the best drug treatment. Specifically, any pharmacological treatment of pain should be scrupulously monitored and carried out in collaboration with experts in the treatment of substance use disorder. Thus, the use of drugs that create a high potential risk of developing addiction, such as benzodiazepines, should be limited or carefully monitored. Last but not least, the possible impact of hormone replacement therapy in this subgroup of patients should be taken into consideration. Up to 20 percent of bipolar women experience a worsening of their symptoms, especially depression, during and after menopause, so highlighting a high sensitivity to hormonal imbalances. Supplemental hormones are sometimes effective in reducing or eliminating the pain linked to endometriosis. The rise and fall of hormones during the menstrual cycle causes endometrial implants to thicken, break down and bleed. Hormone medication may slow endometrial tissue growth and prevent new implants of endometrial tissue. Therapies used to treat endometriosis include hormonal contraceptives (birth control pills, patches and vaginal rings), gonadotropin-releasing hormone (Gn-RH) agonists and antagonists, progestin therapy (intrauterine device with levonorgestrel, contraceptive implants, contraceptive injection or progestin pills), as well as aromatase inhibitors. Several authors showed that hormone medication was associated with increased emotional dysregulation and affective instability [92,107,144,145,146,147]. It can be speculated that women with endometriosis in comorbidity with Bipolar Disorder, and those who are using hormone medication to manage symptoms of endometriosis, are more likely to have to struggle with mood changes and mood disorder symptoms. The administration of hormone therapy makes monitoring imperative in this subpopulation of patients.

The findings of this study reaffirm the importance of providing multi-professional healthcare to women with endometriosis, in view of the social, emotional and physical manifestations of the disease.

### 2.3. Anxiety Disorders, Panic-Agoraphobic Spectrum Disorders and Endometriosis

Most studies have reported a greater tendency in women who have endometriosis to exhibit fluctuations in anxiety levels, panic-agoraphobic spectrum symptoms, an increased stress perception and an elevated degree of stress loading. 

Endometriotic women had significantly more pain, stress, anxiety levels and negative attitudes to menstruation than women who had chronic migraine headaches. This study presents several limitations: the online recruitment caused a non-normal distribution of socioeconomic status of the sample; the lack of a psychiatric examination, with the inevitable result that the identification of psychopathological symptoms is severely limited; the non-exclusion of women with possible overlap of migraines and endometriosis and the eventual impact of other medical comorbidities and drug treatments was not considered [148]. Similarly, women with endometriosis and CPP of moderate intensity turned out to have higher levels of perceived stress, a poorer quality of life and low concentrations of salivary cortisol [149]. Furthermore, in a different study, the same authors showed that endometriotic women with CPP involved in a psychological therapy achieved a significant reduction in perceived stress levels, an improvement in vitality and in physical functioning, besides the normalization of cortisol levels. It would have been very stimulating to perform a psychiatric clinical examination of the patients selected in order to investigate any possible symptoms or psychiatric conditions [77]. Analogously, other authors described increased stress perception in women who had reported reduced quality of life, independently of a diagnosis of endometriosis [109], high prevalence of anxious symptoms as well as an elevated degree of stress loading in endometriotic patients suffering from CPP [80,110,114].

Garalejic et al. (2011), in a sample of patients undergoing laparoscopy for unexplained infertility, described how endometriotic patients reported higher HAM-A scores both before and after the surgical operation compared to those without endometriosis, but the differences were not statistically significant [150]. The lack of a psychiatric evaluation as well as the not identification of the possible symptoms reported by participants limit the results.

A retrospective review of medical records analyzed 138 adolescents/young women (<24 years) who had received a surgical diagnosis of endometriosis noted that patients with comorbid pain syndromes were more likely to report anxiety fluctuations [78]. Additionally, in women experiencing persistent postsurgical pain after surgery for endometriosis, the McGill affective pain score was negatively correlated with age and positively correlated with catastrophism (52% of the sample reported a history of depression and/or anxiety) [123]. In another study, unless no significant difference was observed between women with and without endometriosis regarding the presence of a specific psychiatric syndrome, grouping the disorders in categories, the frequency of anxiety disorders (Generalized anxiety disorder, Panic disorder, agoraphobia, Specific phobia, Social phobia, Anxiety disorder NOS) was significantly higher in women with surgically confirmed endometriosis compared to those without clinical and ultrasound signs of endometriosis, 29% and 7% respectively [83].

Similarly, other studies, described in the previous paragraphs, reported that women with endometriosis, as compared with a healthy control group, presented significantly more pain catastrophism, depression, anxiety symptoms, impairment of sexual functioning, impairment of quality of life, frequent pain during intercourse, higher levels of chronic pain and, further, ran an increased risk of developing anxiety disorders in later life [85,115,124]. As previously described, one recent study showed how the pain amelioration, independently on treatment (surgical or pharmacological), led to a decrease of the anxiety levels and to an improvement of the life quality [125]. 

In another study, young women with endometriosis (<25 years) reported at least mild anxiety (>79%) and depression (>54%). Almost half (45.8%) had moderate to severe levels of anxiety, and a third (33.4%) had moderate to severe levels of depression. In addition, associations between quality of life and maladaptive coping strategies (e.g., autocriticism, social withdrawal) were revealed. In contrast, cognitive restructuring was identified as an adaptive coping strategy that has a positive impact on the quality of life. Despite the small sample size and the non-perspective nature, this study provides further evidence that endometriotic patients presented a high prevalence of anxious and depressive symptoms which, taken together with the implementation of coping strategies, have an impact on the overall well-being of a young patient population; it also identifies opportunities for psychotherapeutic and/or psychopharmacological interventions [151]. 

Finally, one study, unlike the other ones previously mentioned, showed no significant differences between endometriotic women with chronic pain and endometriotic pain-free women in the anxiety dimension. Significant positive correlations were, however, found between coping and anxiety, and between pain severity and subjective psychosocial impairment [66].

Despite the several limitations that are usually recorded, most of these studies highlight a high prevalence of women with endometriosis who present anxiety fluctuations, symptoms of the anxious and panic-agoraphobic spectrum and greater perceived stress. Furthermore, anxiety levels are strongly related to the quality of life, the presence of pain and the severity of depressive symptoms. In this case too we dispose of too few data on the impact that psychotherapy and psychopharmacological treatment have. Putting it simply, it is worth mentioning in this context the risk that endometriotic women with anxious symptoms may come under unrelenting pressure to develop a substance use disorder. These women tend to self-medicate themselves with potentially addictive substances for anxiolytic purposes; this applies especially to BDZs, alcohol, cannabinoids and opioids. In line with the self-medication hypothesis, each individual’s choice of a specific substance is no accident or coincidence, but the outcome of that person’s psychological condition, as the drug of choice provides relief to the user in a way that is specific to his or her condition. Going into greater detail, addiction is hypothesized to function as a compensatory means to modulate effects and treat distressful psychological states, whereby individuals choose the drug that will best respond to their specific type of psychiatric distress and help them achieve emotional stability [152,153,154]. It has been hypothesized that the consumption of substances has the aim of counteracting feelings of isolation and a lack of social relations. From this viewpoint, drugs can be used to substitute these relations or simply produce a feeling of tranquility in social situations. 

Finally, to have a broader view of the paragraph, it is essential to dwell on the complex relationship between anxiety and pain perception. Chronic pain is very frequently associated with anxiety fluctuations. Pain in itself is a strong signal of danger, a sign of an organic "malfunction" that requires attention. The pain activates the nervous system which triggers an alarm signal ("fight or flight"). Collaterally, the activation of the nervous system leads to a greater reactivity of the body with an increase in contractility and muscle tension, heart rate, blood pressure, improves the ability to focus, attention, memorization as well as the onset of feelings of alarm, apprehension, distress and a tendency to worry and catastrophize [155]. While these responses are very helpful in dealing with potentially acute adverse situations, chronically they result into hyperarousal, anxiety, tension, agitation and avoidance behaviors. In patients with chronic pain, these symptoms tend to progressively become chronic, pathological and interfere with overall functioning. Furthermore, the chronic sense of alarm and distress destabilizes the affective state of the patients, increasing irritability and nervousness, as well as the control of emotions. Cognitively, it deteriorates into a chronic focus on pain, which dominates the attention of the pain sufferer. Daily decisions seem to turn on how much pain the patient has to face at any given time. This state of alertness in turn leads to greater chronic muscle tension which amplifies the perception of pain. Lastly, chronic avoidance behaviors lead to a growing sense of social isolation, inactivity, loss of tone and, ultimately, disability [155]. 

The common denominator between chronic pain and chronic anxiety is the hyperactivation of the nervous system.

This persistent state of hyper-reactivity of the nervous system causes the so-called “central sensitization” [156,157,158,159]. 

It is, at least in part, the process by which acute pain becomes chronic pain [70,160,161,162]. 

To sum up, correct management of the anxious dimension may improve patients’ quality of life, pain management and overall outcome.

### 2.4. Temperaments, Personality Traits and Endometriosis

It was Cloninger who defined personality as being divided into two distinct psychobiological dimensions: temperament and character. According to this theory, known as ‘biopsychosocial’, the temperament reflects a biological and hereditary basic structure that determines a “primum” push to act; human character, on the other hand, is interpreted by Cloninger as the result of the individual’s interaction with the environment, in relation to his/her attitudes. Temperament is therefore perceived as the hereditary core of the personality, being stable and relatively unchangeable throughout life, since it has inherited biological characteristics. It determines the basic level of reactivity, mood and energy of any individual. In contrast, the character of an individual strongly depends on the influence that the environment exerts during his/her childhood and adolescence and is therefore linked to the history presented and the cultural heritage learned during individual development. As viewed by Cloninger, therefore, personality is the combination of temperament and character, so it is to be considered a typically dynamic concept within a person’s lifespan [163].

Based on Kraepelin’s concept of temperament, as strengthened by clinical observations, four types of temperament were originally proposed: hyperthymic, depressed, irritable and cyclothymic; the anxious temperament was added later [164,165]. Relationships have been found between Cloninger’s and Akiskal’s selected temperaments [166].

One of the first pertinent studies to be published demonstrated a few differences in personality characteristics between women with endometriosis associated to dysmenorrhea and women who had endometriosis without suffering from dysmenorrhea. The personality traits were assessed using the Rosenzweig picture frustration study—a test of latent hostility [167]. Women with dysmenorrhea tended to express pain in an exaggerated way despite the presence of objective situations that generally evoke pain, without making any attempt to solve it. In contrast, women without dysmenorrhea showed lower scores in the ego-defense dimension, they were less self-assertive and had a less self-protective attitude in facing problems. These are women who choose to stay quiet, because they prefer to refrain from expressing pain, or else may have may have felt indifferent to it, rather than reacting to it [168]. As already described, Carey et al (2014), using the McGill affective pain score, showed that younger age and catastrophism are positively correlated with persistent pain after surgery for endometriosis [123]. In another study, catastrophism not only plays an important role in reported pain management and determining its degree of severity, but also stands as a negative predictive factor in finding the best treatment response to the pain associated with endometriosis. In this case, some limitations may have lowered the value of these findings. First, the diagnosis of endometriosis was based on medical records and patient self-reports. Many psychosocial variables (e.g., histories of trauma and abuse, and substance use) have been left out of consideration. No psychological or psychiatric evaluations were carried out in order to identify any personality traits or specific psychopathological symptoms [169]. 

As well as, Low et al (1993) reported that endometriotic women with CPP had higher scores in psychoticism, introversion and anxiety than women with CPP but without endometriosis [120]. 

In a sample of women treated surgically (laparoscopy or laparotomy) for chronic pelvic pain and with a histological diagnosis of endometriosis, it was found a high negative correlation between the temperament trait of endurance and pain intensity ratings. Further, moderate negative correlations with pain intensity or private beliefs about pain control emerged. The ability to respond adequately, even in conditions of extreme distress, which is the essence of endurance, proved to be an important buffer for the intensity of pain as experienced. In addition, intensity ratings appear to depend on a woman’s beliefs about her own ability to control pain, in other words, internal pain control. All these results have clinical implications; in particular, beliefs about internal pain control could be a prime target in psychological interventions to improve personal control over pain and so extend control over the disease and its course. The study presents several limitations: the sample is far from being representative of the entire population of women with endometriosis, as it was small and self-selected; some social and interpersonal variables such as social support or quality of life were ignored not taken into account; some medical variables (severity of endometriosis, or the influence of the pharmacological management of pain and possible comorbidities) were not considered [170]. Other authors described that greater self-esteem, body esteem and emotional self-efficacy were positively correlated with better psychological outcomes; being in a stable relationship was associated with less rumination. Sexual functioning, pregnancy, infertility, cultural differences and gender beliefs were overlooked. The lack of psychiatric evaluation does not allow definitive conclusions to be drawn [171]. The role that personality traits and coping strategies might have in influencing pain experience was described in a mixed-method sequential explanatory study, consisting of a quantitative survey followed by qualitative interviews. It emerged that acute pain experience, fear of its occurrence, its unpredictability, and control difficulties are the main concerns of women with endometriosis. Worry trait characteristics (i.e., the need to check on problems, anticipatory anxiety, intrusive thoughts centering on worry) and maladaptive thoughts such as coping strategies (namely, self-blame, rumination, catastrophism) were common in this sample and seem to affect how pain is experienced. Although interesting findings emerge, it must be considered that the study presented a limited sample size for the qualitative interview. Furthermore, the patients did not undergo a psychiatric examination and, despite reliance on validated rating scales, part of the task of assessing the answers to the battery of questionnaires administered was web-based; in other cases, telephone conversations were used [96].

The studies that have dealt with temperamental and personality traits reported mostly convergent data. In fact, with the exception of a single study [104], only specific common elements were identified. 

The features detected in these studies tend to define a representative personological profile of endometriotic women who come to the attention of the gynecologist. More specifically, they seem to identify women who complain of CPP when it is linked to endometriosis. Most of the elements identified tend to be part of the vast group of traits and elements included in the C personality cluster. This cluster includes personological profiles distinguished by anxious or inhibited behavior, insecurity in acting, low self-esteem, affective or decision-making dependence and fear of separation, a tendency towards control and perfectionism made more intense by rumination. As we have already seen, these features are unusually strongly represented in women with endometriosis, particularly in those who complain about a moderate or a high level of pain. It could be speculated that these personality traits contribute to their unsuccessful struggle to control pain; they reinforce negative thoughts/beliefs and feelings of powerlessness, leading to psychological distress and a more acute experience of pain. Women showing these personality traits are those who tend most to make the chronicity of pain a distinctive feature, so triggering the vicious circle previously described. 

It should be kept in mind that the most frequently detected personological traits and features in patients with substance use disorders belong to of Cluster B of personality disorders and, secondarily, of Cluster C [172,173,174,175,176]. There are, however, discrepancies, and not all studies endorse this ranking. These discrepancies can be accounted for by the type of substance being consumed; the assessment methods or techniques used; the time of the assessment; whether participants were outpatients or inpatients; the issue of poly- or nonconsumption, or even the stability of the disorders [177,178,179]. To explain the association between elements of Cluster C and SUD, two hypotheses have been put forward. One is the self-medication hypothesis previously described, which proposes that individuals discover that substances alleviate or change a series of painful affective states [153,180]. The second hypothesis, known as ‘stress response dampening’, suggests that individuals with high scores in personality traits, such as stress reactivity, sensitivity to anxiety, and neuroticism, are vulnerable to stressful events. These individuals tend to respond to stress with anxiety and mood swings, which can, in their turn, push them towards substance consumption [181].

In conclusion, these women find themselves at greater risk of developing neuropsychiatric disorders of the affective and anxious spectrum, but also in danger of contracting a substance use disorder that could further aggravate their clinical picture. These women should be considered as a special population that deserves priority attention from the medical team that has been assembled for multidisciplinary therapeutic purposes.

### 2.5. Substance Use Disorders and Endometriosis

Alcohol consumption (its quantity, frequency, and pattern) and alcohol-related problems were determined in endometriosis patients (n = 137), patients with other gynecological disorders (n = 91), and normal control subjects (n = 98), by the use of self-administered questionnaires, including the Michigan Alcoholism Screening Test (MAST) and questions regarding the relationship between gynecological symptoms and alcohol intake [182]. Women with endometriosis had higher MAST scores than those of healthy controls, but such scores did not differ from those of patients with other gynecological disorders. Endometriotic patients with higher MAST scores showed a tendency to consume more alcohol. All patients reported raising their alcohol consumption when experiencing gynecological symptoms. This study suggested for the first time that gynecological problems related to endometriosis may be a major medical correlative of alcoholism in women [91]. It would have been interesting analyze the percentage of women suffering from any psychiatric or other substance use disorders.

A retrospective analysis of the Clinformatics Datamart database and of the Optum Clinformatics Data Mart was performed in order to examine opioid use, opioid prescribing patterns and age at which endometriotic patients (aged 18–49 years) were given their first prescription for opioids, compared with matched women controls without endometriosis. The large-scale study sample included 53,847 endometriosis patients and 107,694 patients in the control group. The women in the endometriosis case group proved to be three times more likely than those in the control group, to receive questionnaire details to qualify for an opioid prescription (79.3% vs. 24.2%). In addition, women in the case group were more likely to receive concomitant opioid and benzodiazepines prescriptions (10.1% vs. 3.5%). Similar results were obtained after excluding opioid prescriptions received during a 30-day post-surgery window. Both for endometriosis patients and those in the control group, acetaminophen-hydrocodone bitartrate (43.2% vs. 14.8%) and acetaminophen-oxycodone hydrochloride (36.4 vs. 5.5%) were the opioids most often named in the prescriptions that had been filled demographic characteristics of the two groups, the women with endometriosis also turned out to have statistically higher rates of mental disorders (20.7% vs. 14.6%), anxiety (13.4% vs. 9.2%), and episodic mood disorders (12.9% vs. 8.7%) than similar women without it, of filling in the details required to obtain a prescription for an opioid. Furthermore, the patients who had endometriosis were at greater risk of filling in details to qualify for a longer-term prescription, prescriptions for a higher dose, or a concomitant prescription for a benzodiazepine. The findings of this study must be considered within the context of its limitations. Women who did have endometriosis were identified with a diagnostic code, which may or may not have been the result of rigorous diagnostic procedures such as pelvic examinations, ultrasound scans and laparoscopy. The study also failed to check whether the diagnosis of endometriosis had been surgically confirmed. In addition, although the authors could determine that prescriptions for opioids or benzodiazepines were filled, they could not confirm how the drugs were taken. The study did not consider an eventual use of illicit opioid and did not control for pain status or the severity of pain [183].

Unfortunately, only a few studies have considered or analyzed substance use or abuse in women with endometriosis. As we have already seen, in a majority of cases, the use of these substances is aimed primarily at pain management and, secondly, at the treatment of any possible associated psychopathological symptoms. Benzodiazepines are mostly anxiolytic drugs, but they are indiscriminately prescribed all over the world so that patients incur a relatively high risk of developing an addiction; alcohol on the other hand, according to the ‘self-medication’ theory could be used by these women for anxiolytic purposes rather than recreational or disinhibitory ones.

According to the psychological profile previously described, these women, after being in contact with these substances, could be more prone to develop an addiction [174]. It follows that the impact of the onset of a substance use disorder in an already precarious and vulnerable clinical setting could be devastating. It follows that specialists should be strongly aware of the potential risk of addiction onset by these patients, so it is absolutely necessary to associate a careful psychiatric monitoring with patients in opioid treatment. At the same time, the prescription of anxiolytic psychotropic drugs, notably benzodiazepines, must be carried out only and exclusively by a psychiatrist who, after a careful clinical examination, in the light of the symptomatic picture, will survey the best choices for administration.

### 2.6. Posttraumatic Stress Disorder (PTSD) and Endometriosis

Seng et al. (2006), after using Michigan Medicaid database, claimed that his data explored the patterns of physical comorbidity among women with post-traumatic stress disorder (PTSD). PTSD was strongly associated with a higher risk of endometriosis (OR= 2.7), and dyspareunia (OR= 3.4), but the relationship between these disorders is not yet clear. Nevertheless, the prevalence of PTSD diagnosis in these data (0.5%) is far below the estimated 4.6% rate of PTSD, and many women with PTSD may be in the comparison group. Thus, these results may underestimate the prevalence of PTSD, and the pattern of physical comorbidity associated with PTSD could turn out to be somewhat different [184].

As part of a study designed to evaluate in a headache clinic population the relationship between childhood maltreatment and the prevalence of pain conditions comorbid with migraine, Tietjen et al. (2010) found a prevalence of 15% of endometriotic patients. They also showed that endometriosis was, to a statistically significant degree, associated with physical abuse [185]. In contrast, one study among women undergoing surgery for a variety of gynecological conditions (e.g., pelvic mass, pelvic pain, irregular menses, infertility, tubal ligation) found no association between physical abuse in childhood and endometriosis. Some limitations should be considered: women with a history of surgically confirmed endometriosis were excluded from the study, as were cases of sexual abuse and the possible presence of emotional support systems, whether in childhood or adulthood [186].

Furthermore, women with histologically confirmed endometriosis reported significantly more frequently than healthy pairs a history of sexual abuse (20% vs. 14%), emotional abuse (44% vs. 28%), emotional neglect (50% vs. 42%) and inconsistency experiences (53% vs. 41 %). No statistically significant differences were demonstrated for physical abuse or neglect (31% vs. 26%). Various combinations of experiences of abuse and/or neglect were described significantly more often in women with endometriosis. Violence against the mother of the family, drug abuse within the family, mentally retarded family members, suicidal intentions in the family, and data on family members who had spent time in prison, did not reveal any statistically significant differences in women with endometriosis and healthy women controls. The limits of the study arose from the nature of the design, which included the risk of both “recall bias” and “sampling bias”, a low statistical capability for sub-analyses, and the overlooking of some psychological and psychosocial variables that could have influenced the definitive results of the study. Some control women, for example, may have presented asymptomatic endometriosis, which would have led to an underestimation of the findings; alternatively, the exclusion of pregnant women may have biased the results [187].

Similarly, in subjects exposed to severe physical or sexual abuse the risk of endometriosis was greater than in those who had not experienced traumas. Indeed, there was a 79% increased risk of laparoscopically confirmed endometriosis for women reporting severe chronic abuse of multiple types. Limitations of this study included the following: only women who were undergoing gynecological surgery were included in the study population. In fact, the author points out that in calculating effect estimates, the analysis relied on comparisons between women with endometriosis and women without it, the problem being that the latter were not “healthy” women. Rather, these women without endometriosis had gynecological pathology, including the sequelae of their surgery (fibroids, adhesions or ovarian cysts, some of which had been previously associated with childhood traumas [188].

Activation of the stress-response network lowers pain thresholds in both humans and animals, and related high anxiety levels have been consistently linked with higher pain sensitivity [72]. Physical and psychological traumas, as well as a prior history of anxiety or mood disorder, have been considered significantly predictive of the onset of chronic pain later in life [76,189,190]. This evidence suggests that the pre-existing dysregulation of the nervous system may result in high susceptibility to the development of chronic pain via the development of central sensitization. Independently of clinical comorbidities, traumatic events are directly correlated with the impaired functioning of the hypothalamic-pituitary-adrenal (HPA) axis and a chronic inflammatory state. This is perfectly in line with the view of endometriosis as a chronic inflammatory process centering on unregulated cytokine release [191,192]. Besides the immunological changes it induces, traumas might result in permanent psychological stress in adulthood. Emotional abuse and neglect during childhood are recognized to often induce anxiety disorders and depression later in life, which are conditions closely connected with endometriosis [193,194]. PTSD impairs sexual functioning across multiple domains: desire, arousal, orgasm, activity, and satisfaction [195,196,197,198]. One logical conclusion is that sexual dysfunctions might lead to relationship troubles that have a consequential impact on affective stability and on the individual’s psychic structure.

## 3. Final Remarks

Endometriosis and the possible presence of accompanying CPP or psychiatric symptoms constitutes a multi-faceted and complex condition, distinguished by a desperate need for a new approach to be founded on a diagnostic and treatment perspective. This new approach model for women with endometriosis provides for a transversal and multiple assessment in terms of management and treatment. Indeed, we should consider that certain hormonal or surgical treatments for endometriosis have been consistently associated with undesirable side effects, including the onset or worsening of psychiatric symptoms. Hormonal contraceptives and gonadotropin-releasing hormone (GnRH) agonists were associated with more severe emotional dysregulation and affective instability [92,144,145,146,147,199]. On the other hand, it should be borne in mind that hormonal endometriosis treatment can improve the physical and psychosocial well-being of the patient, and that these benefits would eliminate the potential risk of psychiatric disorders during endometriosis treatment [4]. 

As we have seen, improper management and treatment of painful symptomatology sometimes leads to the onset of addictions to painkillers such as opioids, or to anxiolytics, which are often prescribed with the unintended result of aggravating pain-related anxiety, so worsening the psychopathological clinical picture. Similarly, some psychiatric medications or an inappropriate prescription of psychiatric drugs may change sex hormone levels and cause menstrual irregularities, with the result complicating the picture [200,201]. Understanding and analyzing the complex symptomatological picture derived from the intricate relationship between psychiatric disturbances and endometriosis can be a major challenge to any clinician.

Finally, we should do not forget the impact that endometriosis has on the relational and sexual life of patients. Patients with endometriosis have more than twice the number of sexual dysfunctions, as compared with women without the disease [202]. As noted by us earlier in the present paper, many patients reported a low index of sexual satisfaction, especially women who felt chronic pelvic pain in comorbidity with psychiatric conditions. Endometriosis, especially when it involves deep infiltrating lesions, can hamper the quality of sexual life. Apart from dyspareunia, these patients can be frustrated and emotionally affected due both to pain and endometriosis-associated infertility, leading to sexual pain disorder and desire-arousal disorder [203,204]. As might be expected, male partners suffer too from the consequences of the partner’s discomfort, showing increased rates of psychological distress, with a higher prevalence of sexual difficulties (e.g., erectile dysfunction), and less sexual satisfaction [205,206,207,208]. At the same time, it should never be overlooked that psychopharmacological therapy as well as hormone therapy can in their turn affect the sexual activity of women who have endometriosis [209,210,211]. It follows that gynecological and pain management treatment cannot be separated from a scrupulous psychiatric assessment in terms of clinical efficacy and prognosis.

**Figure 1:**
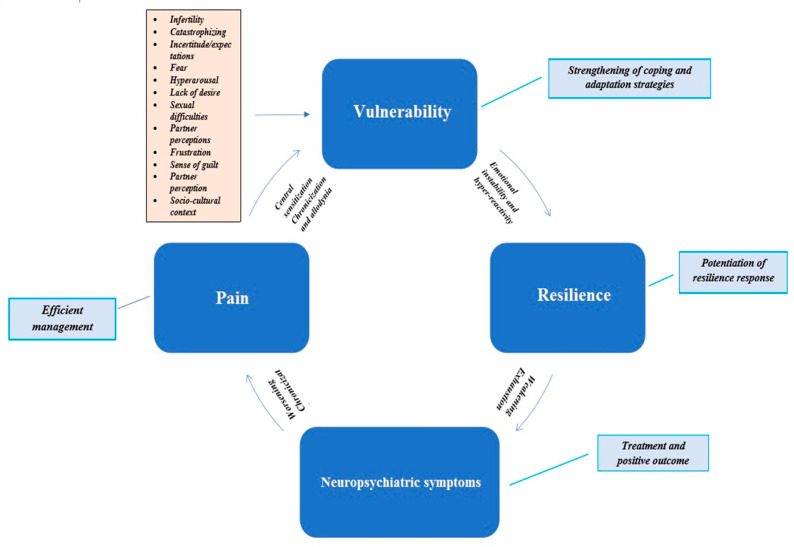
The vicious cycle of Endometriosis.

These observations reaffirm the need for a multidisciplinary team and multiprofessional healthcare, involving gynecologists, psychotherapeutists, psychiatrists, specialists in Dual Disorder, algologists and sexologists. All these professionals should work unidirectionally to detect, analyze and, if possible, break the vicious circle by singling out a customized, targeted treatment (Figure 1). 

Psychological assessment and follow-up must be considered fundamental tools for the management of endometriosis and the improvement of the quality of life of women affected by that disease. Future research policies should address prospective clinical trials to investigate the relationship between endometriosis and psychiatric disorders, with the definitive aim of pressing forward with the task of offering patients personalized, targeted treatments.

## Figures and Tables

**Table 1:** Psychiatric highlights in patients with endometriosis.

Depressive disorder	Elevated rate of women with endometriosis complaining of depressive symptoms, especially those with chronic pelvic pain (CPP) High risk for social, working and functional impairment Marital status has a protective effect Negative correlation with sexual satisfaction Risk of misinterpretation depressive symptoms and consequently setting up an incorrect therapy Psychopharmacological and psychotherapeutic treatment have a positive impact on quality life, pain management and overall outcome
Bipolar disorder	Women with endometriosis have a reactivity to stressors with elevated risk of mood swings The high affective instability reduces the social and working functioning, and quality of life Strongly underestimated and underdiagnosed Mood stabilizers as a fundamental key for a positive overall outcome Not appropriate treatment could cause an acceleration of cycle frequency and an increase of the suicidality High risk of developing a comorbid substance use disorder (licit or illicit substances) Greater attention in the administration of hormone therapy
Anxiety and panic-agoraphobic spectrum disorders	High prevalence of endometriotic patients to present anxiety fluctuations and symptoms of the anxious and panic-agoraphobic spectrum Women with endometriosis and CPP have a greater perceived stress Anxiety levels are strictly related to quality of life, to the presence of pain and to the severity of depressive symptoms Little data about psychotherapy and psychopharmacological treatment High risk of “central sensitization” of pain Risk of develop substance use disorder as a consequence of self-medication drug treatment (Benzodiazepines (BDZs), alcohol, cannabinoids, opioid)
Temperamental and personality traits	Identification of personological profile: anxious or inhibited behavior, insecurity in acting, low self-esteem, affective or decision-making dependence, fear of separation, catastrophizing, tendency towards control and perfectionism Struggle in pain management and risk of “central sensitization” Negative reinforces towards thoughts/beliefs and feeling of powerlessness leading to psychological distress Risk of comorbid affective and/or anxiety disorders Risk of comorbid substance use disorder
Substance use disorder	Worsening of the psychopathological conditions and reduced possibilities to manage the pharmacological treatment Worsening of the pain management Worsening of the sexual satisfaction Risk of relationship problems Reduction of social and working performances High economic expanses Risk of presenting maladaptive behaviors

## Data Availability

Data sharing not applicable: No new data were created or analyzed in this study. Data sharing is not applicable to this article.
